# Hepatic oxidative stress in an animal model of sleep apnoea: effects of different duration of exposure

**DOI:** 10.1186/1476-5926-10-1

**Published:** 2011-07-05

**Authors:** Darlan P Rosa, Denis Martinez, Jaqueline N Picada, Juliane G Semedo, Norma P Marroni

**Affiliations:** 1 Programa de Pós-Graduação em Medicina: Ciências Médicas, Universidade Federal do Rio Grande do Sul, Rio Grande do Sul, Brasil; 2 Programa de Pós-Graduação em Genética e Toxicologia, Universidade Luterana do Brasil, Rio Grande do Sul, Brasil

## Abstract

**Background:**

Repeated apnoea events cause intermittent hypoxia (IH), which alters the function of various systems and produces free radicals and oxidative stress.

**Methods:**

We investigated hepatic oxidative stress in adult mice subjected to intermittent hypoxia, simulating sleep apnoea. Three groups were submitted to 21 days of IH (IH-21), 35 days of IH (IH-35), or 35 days of sham IH. We assessed the oxidative damage to lipids by TBARS and to DNA by comet assay; hepatic tissue inflammation was assessed in HE-stained slides. Antioxidants were gauged by catalase, superoxide dismutase, glutathione peroxidase activity and by total glutathione.

**Results:**

After IH-21, no significant change was observed in hepatic oxidative stress. After IH-35, significant oxidative stress, lipid peroxidation, DNA damage and reduction of endogenous antioxidants were detected.

**Conclusions:**

In an animal model of sleep apnoea, intermittent hypoxia causes liver damage due to oxidative stress after 35 days, but not after 21 days.

## Background

In obstructive sleep apnoea (OSA), pharyngeal occlusion occurs, typically for 10 to 40 seconds, causing a decrease of PaO_2 _and an increase in PaCO_2_, ending with an arousal [1]. Intermittent hypoxia due to OSA causes oxidative stress, a recognized mechanism in the nonalcoholic fatty liver disease (NAFLD), which may progress to nonalcoholic steatohepatitis (NASH) [2].

Intermittent hypoxia (IH) increases liver damage [3]. During hypoxia, activation of xanthine oxidase [4], NAPDH oxidase [5], and phospholipase A_2 _[6] occurs, forming reactive oxygen species (ROS). Increased ROS and decreased antioxidant capacity [7-9] induce oxidative stress [10]. In hypoxia, superoxide anions are formed, which, together with nitric oxide (NO), the main vasodilator, produce peroxynitrite [11-13]. This reaction reduces the bioavailability of NO, attenuating NO-dependent vasodilation, capillary perfusion and expression of adhesion molecules [14-17].

The formation of ROS in OSA is similar to what occurs in ischemia-reperfusion [18]. Oxidative stress leads to inflammation, recognised as a mechanism of the pathophysiology of OSA [19]. Excessive formation of ROS leads to lipid peroxidation in cell membranes, protein oxidation and DNA damage [20-22]. Several ROS are formed in hepatocytes through the activation of Kupffer cells and inflammatory cells [23].

Another group has exposed mice to IH and to a high-cholesterol diet for 6 months, revealing the involvement of OSA in non-alcoholic steatohepatitis (NASH) [3]. IH aggravates paracetamol-induced liver damage after 21 days [24]. To understand the mechanisms leading to NAFLD and NASH it may relevant to identify the time frame in which these phenomena occur. There are, however, no studies specifically investigating the duration of IH exposure that causes liver damage in an animal model of sleep apnoea. This knowledge will be relevant to help design future studies.

The aim of the present study was to establish the duration of exposure to intermittent hypoxia necessary and sufficient to trigger liver damage and oxidative stress in mice.

## Methods

The experimental procedures complied with the rules established by the "Research in Health and Animal Rights" according to the Commission of Research and Ethics in Health of the Research and Postgraduate Group of the Hospital de Clínicas de Porto Alegre.

Thirty-six male CF-1 mice (8-11 weeks old) from Fundação Estadual de Produção e Pesquisa (FEPPS) were employed. They were kept at the Animal Experimentation Unit of the Research Center of the Hospital de Clínicas of Porto Alegre in plastic boxes measuring 30 × 19 × 13 cm lined with wood chips, in a 12-hour dark/light cycle (light from 7 a.m. to 7 p.m.) at a temperature of 22 4°C. The mice were given food (Purina-Nutripal, Porto Alegre, RS, Brazil) and water *ad libitum*.

The animals were randomly divided into three groupings (n = 12): group SIH, sham intermittent hypoxia, which underwent the simulated procedure; group IH-21, exposed to hypoxia for 21 days; and group IH-35, exposed hypoxia for 35 days.

**Figure 1:**
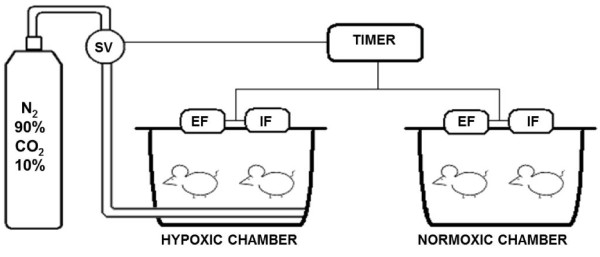
**Diagram of the hypoxic and normoxic chambers**. SV: solenoid valve; EF: exhaust fan; IF: insufflation fan.

IH procedures were described in detail before [25]. In brief, during five weeks, 7 days per week, 8 hours a day, from 9 a.m. to 5 p.m., in the lights-on period, the rodents were placed in the cages (Figure 1). A mixture with 90% nitrogen and 10% CO_2 _was released in the hypoxia chamber, for 30 seconds. The gas mixture reduced the oxygen fraction from 21% to approximately 8% and the CO_2 _fraction to 6%. Subsequently, a fan insufflated room air in the chamber for 30 seconds, restoring the oxygen fraction to 21%. Each hypoxia/normoxia cycle lasted for 60 seconds; in 8 hours, 480 IH periods occurred, equivalent to an apnea index of 60 per hour.

The SIH group was housed in an adjacent cage and underwent the same fan activity as the IH group, but no gas was introduced in the cage during the hypoxia cycle (Figure 1).

On the 21st or 35th day, the animals were killed. They were first anaesthetised with ketamine hydrochloride (100 mg/kg) and xylazine hydrochloride (50 mg/kg ip). Blood was collected from the retro-orbital vein with the aid of a heparinised glass capillary [26] to complete the hepatic integrity (AST, ALT and ALP) test and comet assay. We removed the liver of animals for histological analysis; the rest were frozen -80°C for later biochemical analysis. The animals were euthanized by exsanguination under deep anaesthesia [27,28].

Nine millilitres of phosphate buffer (140 mM KCL, 20 mM phosphate, pH 7.4) per tissue gram was added, and tissue was homogenised in an Ultra Turrax at 4°C. Next, it was centrifuged for 10 minutes at 4,000 rpm (2150.4 g). The samples were stored again at -80°C for posterior analyses.

We used the Bradford method to quantify protein, with bovine albumin as the standard (Sigma^®^). The samples were measured spectrophotometrically at 595 nm, and values expressed in mg/g liver [29] were used to calculate values of TBARS (thiobarbituric acid-reactive substances) and antioxidant enzymes.

The amount of aldehydes generated by lipid peroxidation is measured by the TBARS method, which measures the amount of substances reacting with thiobarbituric acid. The samples were incubated at 100°C for 30 minutes after addition of 0.37% thiobarbituric acid in 15% trichloroacetic acid and centrifuged at 3000 rpm (1612.8 g) for 10 minutes at 4°C. Absorbance was determined spectrophotometrically at 535 nm [30].

The analysis of SOD is based on the inhibition of the reaction of the superoxide radical with adrenaline [31]. The auto-oxidation rate of epinephrine, which is progressively inhibited by increasing amounts of SOD in the homogenate, was monitored spectrophotometrically at 480 nm. The amount of enzyme that inhibited 50% of epinephrine auto-oxidation was defined as 1 U of SOD activity.

The analysis of CAT activity is based on measuring the decrease in hydrogen peroxide [32]. Catalase activity was determined by measuring the decrease in absorption at 240 nm in a reaction medium containing 50 mM phosphate buffer saline (pH 7.2) and 0.3 M hydrogen peroxide. The enzyme activity was assayed spectrophotometrically at 240 nm.

The activity of GPx is based on the consumption of NADPH in the reduction of oxidised glutathione [33]. The glutathione peroxidase activity was determined by the oxidation rate of NADPH in the presence of reduced glutathione and glutathione reductase. Sodium azide was added to inhibit catalase activity. The GPx activity was measured with a spectrophotometer at 340 nm.

Total glutathione (GSH), a water soluble non-enzymatic antioxidant, [34] was measured as described previously [35], in a reaction medium consisting of a solution of 300 mM phosphate buffer (Na2HPO4·1H2O) and a solution of dithionitrobenzoic acid (DTNB). The reaction products were read at 412 nm.

The alkaline comet assay was carried out as described in [36], with minor modifications [37]. The liver tissue samples (200-250 mg) were placed in 0.5 mL of cold phosphate-buffered saline (PBS) and finely minced in order to obtain a cell suspension; the blood samples (50 μL) were placed in 5 μL of anti-coagulant (heparin sodium 25.000 UI- Liquemine^®^). Liver and blood cell suspensions (5 μL) were embedded in 95 μL of 0.75% low melting point agarose (Gilco BRL) and spread on agarose-precoated microated microscope slides. After solidification, slides were placed in lysis buffer (2.5 M NaCl, 100 mM EDTA an 10 mM Tris, pH 10.0), with freshly added 1% Triton X-100 (Sigma) and 10% DMSO for 48 h at 4°C. The slides were subsequently incubated in freshly prepared alkaline buffer (300 mM NaOH and 1 mM EDTA, pH > 13) for 20 min, at 4°C. An electric current of 300 mA and 25 V (0.90 V/cm) was applied for 15 min to perform DNA electrophoresis. The slides were then neutralized (0.4 M Tris, pH 7.5), stained with silver and analyzed using microscope. Images of 100 randomly select cells (50 cells from each of two replicate slides) were analyzed from each animal. Cells were also visually scored according to tail size into five classes ranging from undamaged (0) to maximally damage (4), resulting in a single DNA damage score to each animal, and consequently to each studied group. Therefore, the damage index (DI) can range from 0 (completely undamaged, 100 cells × 0) to 400 (with maximum damage, 100 × 4). Damage frequency (%) was calculated based on the number of tailed versus tailless cells.

The levels of nitrates and nitrites were measured by the reaction of the samples with Griess reagent. Aliquots of 50 μL were incubated with enzyme cofactors and nitrate reductase for 30 minutes at room temperature for the conversion of nitrate to nitrite. The nitrite formed was then analysed by reaction with the Griess reagent, forming a coloured compound that was measured by spectrophotometer at a wavelength of 540 nm [38].

For histological evaluation, part of the liver was preserved in 10% formalin for 24 hours, embedded in paraffin, and cut into 6-μm thick sections with a microtome. Sections were stained with hematoxylin and eosin.

The results are expressed as mean ± standard error. We used ANOVA and the Student-Newmann-Keuls or Student's t-test for comparing groups. The significance level was 5% (p < 0.05).

## Results

The circulating levels of the liver enzymes aspartate aminotransferase (AST), alanine amino transferase (ALT), and alkaline phosphatase (ALP), parameters of liver damage, showed no significant difference between the IH-21 group and the SIH. The IH-35 group showed significantly increased levels (p < 0.05) compared to the sham intermittent hypoxia group (Table 1).

**Table 1:** Enzymes indicating hepatic integrity: AST, ALT and alkaline phosphatase.

Enzymes	SIH	IH-21	IH-35
AST _(U/L)_	124.4 ± 6.5	94.36 ± 7.05	145.8 ± 7.2^a^
ALT _(U/L)_	45.5 ± 4.0	48.50 ± 2.85	55.6 ± 1.3^b^
AP _(U/L)_	97.7 ± 3.1	84.25 ± 1.98	122.6 ± 2.4^c^

Data are presented as mean ± standard error (n = 12 animals/group). ^a ^IH-35 vs SIH, p = 0,04; ^b ^IH-35 vs SIH, p = 0,03; ^c ^IH-35 vs SIH, p < 0,0001. SIH: sham intermittent hypoxia group; IH-21: intermittent hypoxia for 21 days; IH-35: intermittent hypoxia for 35 days; AST: aspartate aminotransferase; ALT: alanine aminotransferase; ALP: alkaline phosphatase.

**Figure 2:**
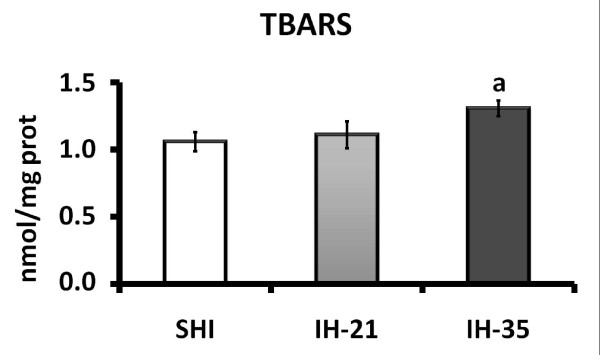
**Effect of intermittent hypoxia on hepatic lipid peroxidation, evaluated using the TBARS assay**. Data are mean ± standard error of the mean (n = 12 animals/group). ^a^, p = 0.0182 vs. SIH. SIH: sham intermittent hypoxia group; IH-21: intermittent hypoxia for 21 days; IH-35: intermittent hypoxia for 35 days.

**Figure 3:**
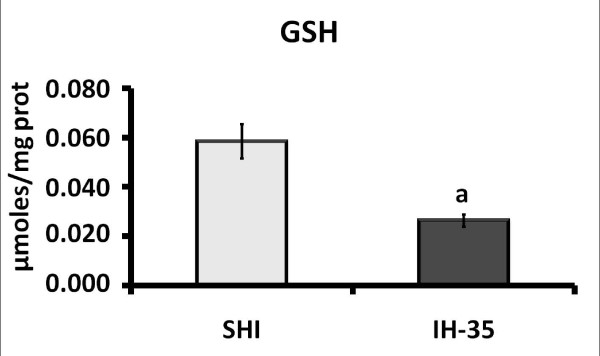
**Effect of intermittent hypoxia on total liver glutathione**. Data are mean ± standard error of the mean (n = 12 animals/group). ^a^, p = 0.0008 vs. SIH. SIH: sham intermittent hypoxia group; IH-35: intermittent hypoxia for 35 days.

Lipid peroxidation measured by the TBARS technique showed no oxidative damage in group IH-21 compared to SIH. However, there was significant damage in the lipid peroxidation in liver subjected to hypoxia for 35 days (Figure 2). Evaluation of the antioxidant enzymes showed a significant decrease in the activities of superoxide dismutase (SOD), glutathione peroxidase (GPx) and catalase (CAT) in liver tissue with intermittent hypoxia for 35 days (Table 2). The quantification of total endogenous glutathione in the liver showed a significant decrease in the 35-day hypoxia group compared with the sham intermittent hypoxia (Figure 3). These results demonstrate that IH induced a decrease in the endogenous antioxidant defence.

**Table 2:** Activities of liver antioxidant enzymes.

Enzymes	SIH	IH-35	p value
SOD _(USOD/mg prot)_	4.63 ± 0.26	3.16 ± 0.25	0.0005
GPx _(mmol/min/mg prot)_	1.00 ± 0.11	0.52 ± 0.06	0.0028
CAT _(pmol/mg prot)_	1.06 ± 0.04	0.79 ± 0.03	0.0003

Data are mean ± standard error (n = 12 animals/group). SIH: sham intermittent hypoxia group; IH-35: intermittent hypoxia for 35 days. SOD: superoxide dismutase; GPx: glutathione peroxidase; CAT: catalase.

The assessment of DNA damage by the comet assay showed that the damage in blood did not differ between groups, but the liver tissue exhibited a significant increase in DNA damage in group IH-35 compared with SIH (Table 3).

**Table 3:** Comet assay on peripheral blood and liver tissues from mice subjected to hypoxia.

	SIH		IH-35	
**Tissue**	**Damage index^a^**	**Damage frequency^b^**	**Damage index**	**Damage frequency**

Blood	15.3 ± 4.4	7.6 ± 1.3	19.3 ± 4.1	8.0 ± 1.4
Liver	38.1 ± 5.1	14.8 ± 1.8	114.7 ± 32.3**	43.2 ± 11.3**

Data are presented as mean ± standard error (n = 6 animals/group). SIH: sham intermittent hypoxia group; IH-35: intermittent hypoxia for 35 days. ^a^, Damage index: can range from 0 (completely undamaged, 100 cells × 0) to 400 (with maximum damage, 100 × 4). ^b^, Damage frequency: calculated based on the number of cells with tails versus those with no tail. **, p < 0.01, statistically significant difference from sham intermittent hypoxia group (t-test).

In the assessment of metabolites of nitric oxide in liver tissue of mice subjected to IH for 35 days, we noted a significant increase in NO in these animals compared with SIH (Table 4).

**Table 4:** Quantification of nitric oxide metabolites in liver tissue.

Metabolites	SIH	IH-35	p value
NO_2(μmol/L)_	2.128 ± 0.202	3.405 ± 0.112	0.0001
NO_3(μmol/L)_	0.018 ± 0.002	0.050 ± 0.003	0.0001

Data are mean ± standard error of the mean (n = 12 animals/group). SIH: sham intermittent hypoxia group; IH-35: intermittent hypoxia for 35 days; NO_2_: total nitrate; NO_3_: nitrites.

**Figure 4:**
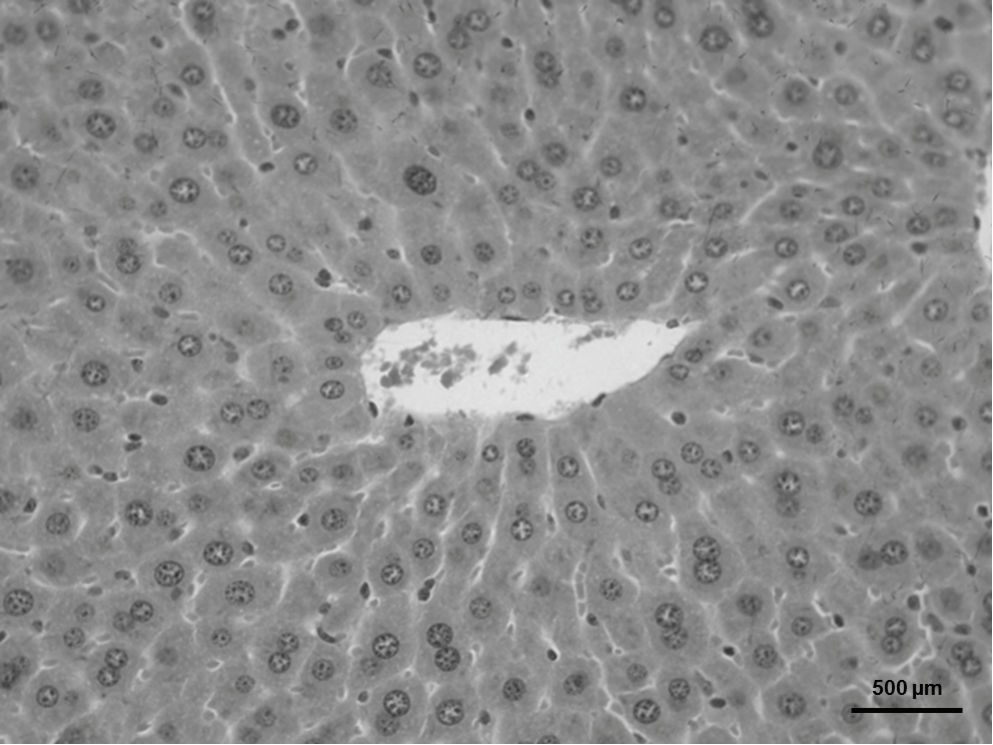
**Photomicrograph of the mouse liver in sham intermittent hypoxia condition**. A normal histological pattern was observed. Hematoxylin and eosin.

**Figure 5:**
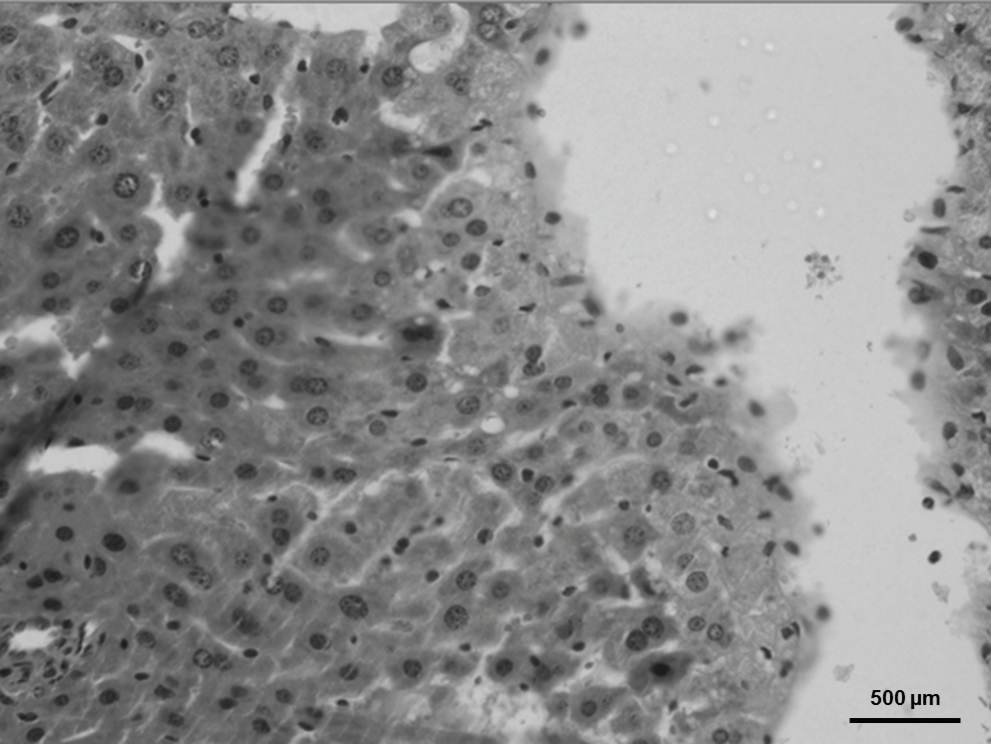
**Photomicrograph of the mouse liver in intermittent hypoxia for 35 days**. It was observed cellular ballooning, steatosis, necrosis and the presence of neutrophils. Hematoxylin and eosin.

Several histological liver changes were also observed in animals of the IH-35 group - ballooning, steatosis, necrosis and the presence of neutrophils -when compared with mice under sham intermittent hypoxia (Figures 4 and 5).

## Discussion

We report for the first time that 35 but not 21 days of exposure to IH, simulating an OSA of 60 events per hour, reducing for 6% the concentration of oxygen, causes hepatic damage. This is also the first report to combine the description of enzyme, lipid, DNA, oxidative, and nitrosative hepatic damage. We used an experimental model that produces levels of hypoxia comparable to those observed in patients with severe OSA [24,39]. Although our findings cannot be immediately translated to the clinical setting, they are in agreement with the literature indicating an OSA-NASH association [40,41].

Two mechanisms are proposed for the morbidity caused by OSA: the activation of inflammatory factors and oxidative stress [42,43], which also can be modulated by genetic, lifestyle and environmental factors [43,44]. Oxidative stress plays an important role in various diseases as well as in OSA, which causes an effect similar to ischemia-reperfusion [18] in which there is activation of xanthine oxidase, leading to the formation free radicals and further imbalance between oxidants and antioxidants [4-6].

The analysis of liver integrity showed that the liver tissue of mice subjected to intermittent hypoxia was damaged, but only after 35 days, as demonstrated by the significant increase in circulating AST, ALT and alkaline phosphatase. The present results demonstrate damage both at cytoplasmic and mitochondrial level, confirmed by the presence in the histological examination of ballooning, steatosis, necrosis and the presence of neutrophils in the liver, similar to what is observed in NASH [45].

In the evaluation of hepatic lipid peroxidation, we observed a significant increase in lipid oxidative damage in animals that were subjected to hypoxia for 35 days, as indicated by the TBARS test, but not in group IH-21. This damage can be caused by the increase of free radicals in the liver tissue. Similar data have been reported in other studies of intermittent hypoxia [46-48] and by our laboratory in other experimental models of hepatic oxidative damage [49-54].

As we did not observe liver damage in animals exposed to IH for 21 days, by the liver enzyme, histological, or lipid peroxidation assays, we concluded that this duration of IH causes no damage to the organ. Therefore, dosages of antioxidant enzymes, comet assay and nitrites metabolites were not conducted in the IH 21 group.

Comet assay in liver tissue revealed a significant increase in DNA damage in the IH-35 group in comparison to the SIH group. No evidence of damage was observed in blood tissue. The rate of DNA damage detected by the comet assay depends on the tissue or organ analyzed [55]. Here, the DNA damage was observed only in the tissue most susceptible to lesions produced by IH. In the alkaline version used, the comet assay detects a broad spectrum of DNA lesions, including single strand breaks [56,57].

Previous comet assay and TBARS data have demonstrated increased formation of free radicals in sleep apnoea patients [11]. Possibly, the formation of superoxide radical (O_2_^-•^) and hydrogen peroxide (H_2_O_2_), which appear to be increased in individuals with OSA, is due to the conversion of xanthine dehydrogenase (type D) into its oxidase (type O) form in hypoxia, followed by the activation of the oxidase form during reoxygenation (normoxia) by the hypoxanthine formed during hypoxia. This xanthine oxidase activity generates O_2_^-•^, H_2_O_2_, and uric acid [4,11].

Our evaluation of the endogenous antioxidant liver enzymes SOD, GPx and CAT showed that their activities were significantly decreased in mice after 35 days under intermittent hypoxia. Quantification of total glutathione revealed significant decreases in the group exposed to intermittent hypoxia compared to SIH, demonstrating a reduced hepatic antioxidant defence in these animals.

The increase in TBARS and decrease in endogenous antioxidants observed in the present study further promotes oxidative stress, contributing to aggravation of the liver tissue injury. This kind of pathological synergy is evidenced in experimental models of liver damage induced by xenobiotic agents that cause oxidative stress such as carbon tetrachloride and toluene [49,50,52,54,58], by surgical procedures such as ligation of the common bile duct [51,53] or by thymoquinone [59].

The increased nitric oxide metabolites nitrite and nitrate in the livers of IH-35 mice confirms findings by other authors, who demonstrated a significant increase of nitric oxide in animals exposed to IH simulating OSA (6 min/6 min) during 120 days [48], and to hypobaric hypoxia during 32 days [60]. The increase of NO, along with increased free radicals, may generate nitrosative stress caused by the reaction products of these two substances, such as peroxide nitrite (OONO^•^) formed by the reaction between NO and O_2_^-• ^[11]. Much evidence indicates that oxidative and nitrosative stress have important roles in the complication of hypoxia [61].

OSA is usually accompanied by arterial hypertension, pulmonary hypertension, myocardial infarction and stroke, which may be due to changes in nitric oxide production [62]. Veasey et al. had demonstrated irreversible basal forebrain nitrosative damage as a possible cause for residual sleepiness in OSA [63].

It is increasingly clear that IH is capable of causing liver tissue damage. This was here demonstrated by several lines of evidence: elevated circulating levels of liver enzymes, NO increase, damage to lipids and DNA, and reduced endogenous antioxidant defences. Further translational research is necessary to completely correlate these findings with the NASH pathology.

## Conclusions

The present results suggest that a model of intermittent hypoxia for 35 days, simulating sleep apnoea, is useful to investigate liver injury by oxidative and nitrosative stress. Exposure to intermittent hypoxia during 21 days may be insufficient to produce hepatic damage.

## Competing interests

The authors declare that they have no competing interests.

## Authors' contributions

DPR conducted the animal studies. DPR and JGS collected tissues and performed analyses. DPR and DM wrote the manuscript. JNP, NPM and DM reviewed the manuscript. DPR and DM designed the study and reviewed the manuscript. All the authors read and approved the final manuscript.
